# Revisiting the cost-effectiveness of universal HPV-vaccination in Denmark accounting for all potentially vaccine preventable HPV-related diseases in males and females

**DOI:** 10.1186/s12962-015-0029-9

**Published:** 2015-02-11

**Authors:** Jens Olsen, Tine Rikke Jørgensen

**Affiliations:** Centre for Applied Health Services Research and Technology Assessment (CAST), University of Southern Denmark, 5000 Odense C, Denmark; Incentive, 2840 Holte, Denmark; Sanofi Pasteur MSD ApS, 2800 Kgs, Lyngby, Denmark; Medivir, 2800 Kgs, Lyngby, Denmark

**Keywords:** HPV-vaccination, Cost-effectiveness, HPV-related diseases, Cancer, Genital warts, Gender neutral vaccination

## Abstract

**Objective:**

The purpose of this study was to assess the consequences of a national immunization program with HPV vaccine for both boys and girls in Denmark, including the prophylactic effects on all potentially vaccine preventable HPV-associated diseases in male and female.

**Methods:**

The study focussed on the quadrivalent vaccine which protects against HPV type 6, 11, 16 and 18, and the vaccine’s protection against genital warts, cervical intraepithelial neoplasia, cervical cancer, anogenital cancer (anal, penile, vaginal and vulvar cancer) and head and neck cancer (oral cavity, oropharyngeal, hypopharyngeal and laryngeal cancer) were included in the analyses. In general, the analysis was performed in two phases. First, an agent-based transmission model that described the HPV transmission without and with HPV vaccination was applied. Second, an analysis of the incremental costs and effects was performed. The model did not include naturally-acquired immunity to HPV in the simulations.

**Results:**

In the base case result (i.e. vaccination of girls only, 85% vaccination rate, private market price at € 123 per dose ex. VAT) an ICER of 3583 €/QALY (3-dose regime) is estimated when all HPV-related diseases are taken into account. Vaccination of girls & boys vs. vaccination of girls only an ICER of 28,031 €/QALY (2-dose regime) and 41,636 €/QALY (3-dose regime) is estimated.

**Conclusions:**

Extension of the current HPV programme in Denmark to include boys and girls is a cost effective preventive intervention that would lead to a faster prevention of cancers, cancer precursors and genital warts in men and women.

## Introduction

Persistent human papillomavirus (HPV) infections with HPV genotype 16 and genotype 18 are responsible for about 70% of all cervical cancer [1-3]. HPV causes not only cervical cancer, but is also accounting for 40-85% of cases of anal, penile, vaginal, and vulvar cancer. Not only anogenital cancer is linked to HPV, but also some head and neck cancers, as 16-28% of cancers of oral cavity, oropharyngeal, hypopharyngeal, and laryngeal are attributable to HPV [4]. HPV 16 and HPV 18 cause most of the HPV linked anogenital cancer and head and neck cancers, as HPV 16 or 18 are accounting for 74-100% of all these cancers [5-11]. HPV is a cause of not only cancer, the HPV types HPV 6 and 11 cause about 90% of anogenital warts [12]. In addition juvenile onset of recurrent respiratory papillomatosis is also caused by HPV types 6 and 11 [13].

Two vaccines are currently available on the market: a quadrivalent (including HPV genotypes 16, 18, 6 and 11) and a bivalent vaccine (including genotypes 16 and 18). Both vaccines effectively protect against precancerous lesions in the cervix, vulva or vagina and cervical cancer; in addition, the quadrivalent prevents precancerous anal lesions, anal cancer and anogenital warts.

Currently, HPV vaccination of adolescent girls is part of the national immunization programme in a number of countries [14-16]. In the past 5-6 years, numerous cost-effectiveness studies of HPV-vaccination evaluating various vaccination scenarios have been published [14,16-23]. However, few studies include the prophylactic effect of all HPV-associated diseases [18,19,22], despite the considerable burden of non-cervical HPV disease – especially in men [18,24,25]. This applies e.g. in Denmark, where the burden of HPV-linked cancer, in particular HPV 16 linked head and neck cancer, is higher in men than in women [25]. In recent years, there has been a moderate decrease in the cervical cancer incidence in Denmark whereas the incidence in head and neck cancer has been increasing. In 2012, the incidence rate (i.e. age adjusted incidence per 100,000) for cervical cancer was 12.7, the incidence rate for anogenital cancer (i.e. anal, penile (in men), vaginal (in women), and vulvar (in women) cancer) was 6.4 in women and 3.6 in men and the incidence rate for head and neck cancer (i.e. cancers of oral cavity, oropharyngeal, hypopharyngeal, and laryngeal) was 8.9 in women and 19.6 in men [26].

Since 2009, the national immunization programme in Denmark offers free quadrivalent human papillomavirus vaccine to all girls aged 12 years. Furthermore, in 2012 and 2013 a catch up program was implemented for all women up to 27 years old.

The purpose of this study was to assess by modelling the economic consequences of a national immunization program with publicly financed quadrivalent HPV vaccine for both boys and girls aged 12 years or a for only girls aged 12 years in Denmark compared with screening program alone, including the prophylactic effects on all HPV-associated diseases.

## Methods

The following scenarios were analysed in the study:

- the cost-effectiveness of vaccinating 12-year-old girls in Denmark compared to no HPV vaccination

and

- the cost-effectiveness of vaccinating 12-year-old girls and boys in Denmark compared to vaccinating girls alone.

The study focussed on the quadrivalent vaccine which protects against HPV type 6, 11, 16 and 18, and the vaccine’s protection against genital warts, cervical intraepithelial neoplasia, cervical cancer, anogenital cancer (anal, penile, vaginal and vulvar cancer) and head and neck cancer (oral cavity, oropharyngeal, hypopharyngeal and laryngeal cancer) were included in the analyses. Protection against recurrent respiratory papillomatosis was not included in the analysis among others because no Danish study reports the costs of recurrent respiratory papillomatosis.

### Model simulations and assumptions

A previously developed and published model was used [15,27]. This model has also been applied in an Irish setting by the national authorities [28].

The analysis was performed in two phases. First, using the software NetLogo (version 4.0.2) (http://ccl.northwestern.edu/netlogo), an agent based transmission model was developed that describes the HPV transmission dynamics before and after introduction of HPV vaccination. Second, an analysis of the incremental costs and effects was performed using Microsoft® Excel. A more comprehensive description can be found in [15] and the original Health Technology Assessment [27] includes an exhaustive description (20 pages) of the model.

In the agent based model the transmission and possible clearance of HPV 6, 11, 16 and 18 was simulated in a heterosexual population and for persistent HPV infections, the subsequent development of genital warts, cervical intraepithelial neoplasia (CIN1–3) and cervical cancer were also simulated. No natural immunity following infection was assumed, suggesting that an individual infected by one type of HPV has the same risk of being re-infected by the same type.

The acquisition of HPV infection and natural course of HPV related cancers at non-cervical sites are poorly understood. Hence, it is difficult to construct a model of HPV infection and disease in these sites that accurately captures the underlying biological processes [29]. As a simplification, it was assumed that the incidence of HPV attributable to non-cervical cancers will decrease at the same rate (proportionately) as cervical cancer, as predicted by the transmission model.

The model operates with several variables, some of which relate to the entire population (e.g. CIN progression and regression probabilities, risk of HPV infection per sexual act) and some of which are agent-specific (e.g. age, sex, duration of relationship), see Table 1.

**Table 1:** **Parameters applied in the HPV transmission model**

**Variable**	**Value**	**Source**
Age groups included	10-78 years, present Danish age distribution	Age distribution: Statistics Denmark
Gender	Man/woman	Fixed at 50%
Concurrent partners	0, 1 or 2, uniform/block distribution	Estimate.
Duration of relationship (in months)	Based on estimate, dependent of age, that is the older the longer duration (Y = abs random-normal (0.8·age – 12) (age/0.5)·12).	Estimate
Frequency of sexual intercourse	Random-gamma distribution with a mean of 9.48 per month; SD 9.95	Burchell et al. [30]
Vaccination status	0 or 1	
HPV-specific:		
Duration of HPV 6 infection (in months)	Exponential distribution with a mean of 11 months	The estimate is set in order to calibrate the model before introduction of a vaccine
Duration of HPV 11 infection (in months)	Exponential distribution with a mean of 9.5 months	
Duration of HPV 16 infection (in months)	Exponential distribution with a mean of 13 months	
Duration of HPV 18 infection (in months)	Exponential distribution with a mean of 11 months	
HPV → CIN1	0.0049 per month (probability)	
CIN1 → HPV/clear (regress)	0.329 per year (probability)	Elbasha et al. [17]
CIN1 → CIN2	0.46 per year (probability)	The estimate is set in order to calibrate the model before introduction of a vaccine
CIN2 → CIN1 (regress)	0.1 per year (probability)	
CIN2 → CIN3	0.60 per year (probability)	
CIN3 → CIN2 (regress)	0.02 per year (probability)	
CIN3 → cervical cancer	0.37 per year (probability)	
HPV 6/11 → genital warts	0.30 per year (probability)	
Risk of infection		
−for HPV 6/11/16 per intercourse	0.3	Elbasha et al. [17]
−for HPV 18 per intercourse	0.13	Modified compared to the HPV 16 risk to take into account a lower HPV 18 prevalence
Vaccine efficacy	100%	

It was assumed that the current Danish cervical screening program remained unchanged implying that women aged 23-64 years are offered screening every 3-5 years. Therefore, cervical cancer screening was not modelled. A 62-year time horizon was applied implying that the first year group of 12-year-olds are followed until the age of 74 years in order to include all costs and benefits of the vaccination programme (i.e. the second year group of 12-year-olds are followed 61 years etc.).

The vaccine’s possible protection against non-vaccine HPV types (i.e. cross protection) was not included in the analyses even though slight evidence suggests that some cross protection might exist (SPC Gardasil and Cervarix, http://www.ema.europa.eu/ema). In some studies cross protection are included (e.g. [29]). Thus, cross protection in this analysis implies that equal cross protective efficacy for the two existing vaccines were assumed.

Compared to the previous publication [15], the model has been adjusted with updated data on cancer incidence and mortality.

### Cost and effects

For each scenario the incremental cost (vaccination cost minus future costs averted by vaccination) per life year gained (LYG) and the incremental cost per quality adjusted life year (QALY) gained by the HPV-vaccination was estimated. Given the age distribution for cancer deaths and that for the general population, it was possible to estimate the life-years gained (LYG) due to a lower incidence of cervical cancer, anogenital cancer and head and neck cancer. Similarly, given the modelled decrease in the specific diseases and given the QALY-weights for the general population and the specific diseases (Table 2), the gain in QALYs was estimated.

**Table 2:** **QALY weights applied**

**QALY weights the general population** ^**1**^					
	**Men**	**Women**		**QALY**	
Age group	QALY	QALY	Occurrence/illness	Utility score^2^	Duration
15-19	0.9373	0.9203			
20-24	0.9373	0.9203	CIN1	0.93	2 months
25-29	0.9373	0.9203			
30-34	0.9355	0.9118	CIN2-3	0.87	2 months
35-39	0.9197	0.8898			
40-44	0.9118	0.8763	Genital warts	0.91	85 days
45-49	0.9050	0.8751			
50-54	0.8813	0.8499	Cervical cancer, fatal	0.76-0, 67	
55-59	0.8870	0.8542	Cervical cancer, survivors	0.76	5 years
60-64	0.8747	0.8552			
65-69	0.8801	0.8098	Genital cancer, fatal	0.76-0.67	
70-74	0.8437	0.8320	Genital cancer, survivors	0.76	5 years
75-79	0.8429	0.7837			
80-84	0.7855	0.6919	Head & neck cancer, fatal	0.76-0.67	
85-	0.7855	0.6919	Head & neck cancer, survivors	0.76	5 years

^1^Unpublished data from survey performed by National Institute of Public Health, University of Southern Denmark (personal communication). These data, pooled with two other Danish surveys, are published on a more aggregate level in Sørensen et al., 2009 [31].

^2^Source: [18].

A health care sector perspective was applied and included direct costs of vaccination and future averted health care sector costs of HPV-associated diseases. The applied health care costs appear from Table 3.

**Table 3:** **Cost estimates**

	**Cost estimate (PV)**	**Source**
Vaccination cost	417 €	Cost estimates in [15] updated to 2008 price level
Treatment costs – genital warts	247 €	
Treatment/control CIN1 and atypia	34 €	
Treatment costs CIN2-3	2,780 €	
Treatment costs cervical cancer	25,546 €	
Treatment costs genital cancer	24,640 €	Weighted average based on [24]
Treatment costs head & neck cancer	30,400 €	Weighted average based on [25]

2008 price level. PV: present value. Applied discount rate: 3%.

Future costs and effects were discounted at a rate of 3% in the base case.

### Sensitivity analyses

One-way sensitivity analyses varying one parameter, while holding other parameters at their base case value, were performed. The parameters varied appear from Table 4. Vaccination rate refer to the 3-dose coverage of 12-year-old girls (and boys if applicable).

**Table 4:** **Results: Cost-effectiveness of HPV-vaccination in Denmark including protection against genital cancer and head & neck cancer**

	**Average incremental vaccination costs per year (€, PV)**	**Average savings in treatment costs per year (€, PV)**	**Average incremental cost per year (€, PV)**	**Average LYG (PV)**	**Average QALYs gained (PV)**	**ICER (€/LYG)**	**ICER (€/QALY)**	**Average number of cases avoided per year**
Vaccinations of girls vs. screening alone:								
Base case result*	11,504,613	9,480,325	2,024,288	528.5	565.3	3,830	3,581	
Cervical cancer								197
Anogenital cancer								80
Head and neck cancer								43
Univariate sensitivity analysis:								
Discount rate 0%	11,504,613	19,622,317	- 8,117,704	1,706.5	1,645.5	Dominance	Dominance	
Discount rate 5%	11,504,613	6,603,582	4,901,031	265.3	312.5	18,476	15,682	
Costs discounted with 3% and LYG & QALYs with 0%	11,504,613	9,480,325	2,024,288	1,706.5	1,645.5	1,186	1,230	
Vaccine price reduced by 25%	8,967,267	9,480,325	−513,058	528.5	565.3	Dominance	Dominance	
2-dose regime	8,121,485	9,480,325	−1,358,840	528.5	565.3	Dominance	Dominance	
Treatment cost reduced by 25%	11,504,613	7,055,464	4,449,149	528.5	565.3	8,418	7,870	
Time horizon 40 years	11,504,613	8,450,127	3,054,486	425.2	472.2	7,184	6,469	
Bivalent HPV vaccination (HPV types 16 & 18)	11,504,613	5,269,690	6,234,923	528.5	505.1	11,797	12,345	
Protection against head and neck cancer excluded:	11,504,613	9,159,308	2,345,305	432.1	470.4	5,428	4,985	
70% vaccination rate	9,474,387	9,320,914	153,473	489.2	551.9	314	278	
Cervical cancer								192
Anogenital cancer								77
Head and neck cancer								42
Vaccination of girls & boys vs. girls:								
Base case result**	11,858,601	1,186,760	10,671,841	263	256	40,615	41,636	
Cervical cancer								5
Anogenital cancer								34
Head and neck cancer								98
Univariate sensitivity analysis:								
Discount rate 0%	11,858,601	4,298,652	7,559,950	889	863	8,500	8,763	
Discount rate 5%	11,858,601	587,457	11,271,144	124	122	90,704	92,015	
Vaccine price reduced by 25%	9,243,183	1,186,760	8,056,423	263	256	30,661	31,432	
2-dose regime	8,371,377	1,186,760	7,184,617	263	256	27,343	28,031	
Time horizon 40 years	11,858,601	1,194,383	10,664,218	239	225	44,674	47,342	
Protection against head and neck cancer excluded	1,858,601	459,212	11,399,389	42	41	269,857	276,642	
70% vaccination rate	9,765,907	1,281,863	8,484,044	301	268	28,146	31,615	

2008 price level.

*Vaccination over 62 years (implying that the incremental costs and effects are calculated as an average over the 62 years, assuming vaccination from the first year and onward) of 12-year-old girls compared to screening alone, 85% vaccination rate, discount rate 3%, 100% vaccine efficacy, vaccine price: € 123 per dose ex. VAT (=market price, source www.promedicin.dk, 10th of May, 2012).

**Vaccination over 62 years of 12-year-old girls and boys compared to 12-year-old girls alone, 85% vaccination rate, discount rate 3%, 100% vaccine efficacy, vaccine price: € 123 per dose ex. VAT (=market price, source www.promedicin.dk, 10th of May, 2012).

PV: present value; QALY: quality-adjusted life-year; ICER: incremental cost-effectiveness ratio; LYG: life-year gained.

**Figure 1:**
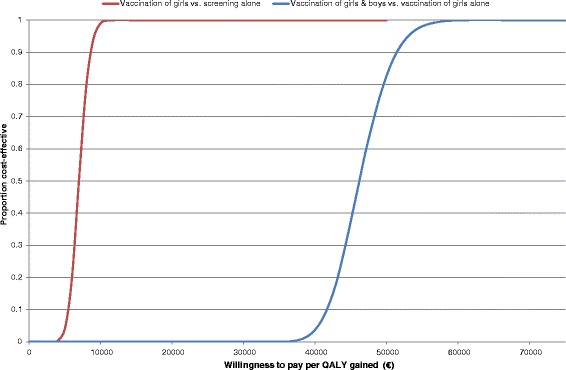
**Cost-effectiveness acceptability curves: proportion of simulations that would be cost-effective given different thresholds values of cost per QALY gained.** It should be noted that this multi-way sensitivity analysis did not address all sources of uncertainty as some parameters were not included.

Based on a recently published study, Danish authorities have changed the quadrivalent vaccine regime from 3 doses to 2 doses [32]. Therefore, a sensitivity analysis with a 2-dose regime was also perfomed (cf. Table 4). Furthermore, in order to handle the parameter uncertainty multi-way sensitivity analysis was performed. 10,000 model simulations were conducted and in each simulation the following parameters were randomly assigned to their base case value or an increased/decreased value: vaccination rate (70% or 85%), vaccine price (market price or market price reduced with 25%), treatment cost (base case value or reduced with 25%), discount rate (3% or 5%) and time horizon (62 years and 40 years). The results of the multi-way sensitivity analysis were reported as cost-effectiveness acceptability curves (Figure 1).

It should be noted that this multi-way sensitivity analysis did not address all sources of uncertainty as some parameters were not included (e.g. duration of relationship, frequency of sexual intercourse). However, these parameters were varied in the NetLogo simulations as distribution for these parameters were assumed (cf. Table 1).

## Results

Simulations before the introduction of vaccination showed that the estimated HPV prevalence replicated the Danish HPV prevalence (data not shown) fairly precise, indicating that the model was well calibrated (cf. [15]). When vaccination was introduced, the prevalence of HPV 6, 11, 16 and 18 decreased and the rate of decrease among others depends on the applied time horizon. Given a 62-year time horizon, the incidence of e.g. cervical cancer is reduced with 74-77% depending on the vaccination program and vaccination rate. A 40-year time horizon leads to an estimated reduction in the incidence of cervical cancer on 59%.

In the base case result with a 85% vaccination rate the cost per QALY gained by female vaccination (compared to no vaccination) was € 3,581 when including all HPV-related diseases in the analysis (Table 4). A 85% vaccination rate is the relavant base case as recently updated Danish HPV vaccination rate report coverages on 81-90% for 3 doses [33]. Compared to this the cost per QALY gained when only protection against cervical cancer and precancerous lesion are included in the analysis is € 20,644 (result not shown in Table 4).

From Table 4 it is seen that the incremental cost-effectiveness ratios (ICERs) increases with increasing discount rate, reduced treatment cost, reduced time horizon and exclusion of protection against head and neck cancer whereas the ICERs decreases with reduced discount rate, reduced vaccination rate, reduced vaccine price and application of a 2-dose regime. Sensitivity analysis on reduced vaccine price is relevant as the vaccine may be offered at a lower price to the health authorities in a public financed programme. Given a 85% vaccination rate and a 25% reduction in the vaccine price or application of a 2-dose regime the average annual savings in treatment costs outweigh the average incremental vaccination costs per year, making the vaccination program dominant.

Inclusion of the vaccine’s protection against anogenital cancer and head and neck cancer, (the latter being more prevalent in men), in the analysis may better address the relevance of routine vaccination of boys. ICERs of vaccination of girls and boys compared to vaccinaition of girls only appear from Table 4. Compared to vaccination of girls alone the ICERs are higher and again the ICERs increases with increasing discount rate, time horizon and exclusion of protection against head and neck cancer while the ICERs decreases with reduced discount rate, reduced price, application of a 2-dose regime and reduced vaccination rate.

In Denmark, routine vaccination of girls has been conducted since 2008. Currently some countries have expanded the HPV immunization programme to include routine vaccination of boys [34]. Therefore, modelling of universal vaccination of boys and girls compared to vaccination of girls alone may be relevant in a Danish setting. The ICERs are higher implying that it may be beneficial to include boys in the vaccination program as well but the additional effect is decreasing as boys to a certain degree already are protected in a female vaccination program due to herd protection. On the other hand, given a cost-effectiveness threshold on 50,000 € per QALY gained vaccination of boys and girls is estimated to be cost-effective. Vaccinating boys, to prevent diseases that affect women only (cervical, vulvar, and vaginal cancer), lead to relative high ICERs but when all HPV-related diseases are included (penile cancer, anal cancer and head and neck cancer) the ICERs for vaccination of boys and girls versus vaccination of girls alone dropped markedly.

The uncertainty in the results of the cost-effectiveness analysis is summarized in the cost-effectiveness acceptability curves (Figure 1). In Denmark, there is no defined threshold values of cost per QALY gained but it is seen from Figure 1 that vaccinating girls is cost-effective even at relative low thresholds values.

## Discussion

This study was based on a Danish agent based model on HPV transmission and vaccination. The model was initially developed for the Danish National Board of Health [27]. This study modelled all HPV related diseases in males and females as opposed to previous studies focussing primarily on prevention of HPV related cervical cancers including precursors for cervical cancers. Denmark has a relatively high incidence of HPV related diseases in men and women and potential routine vaccination of girls and boys has continuously been discussed after the publication of a Danish Health Technology Assessment in 2007 [27]. In addition, several countries recommend HPV vaccination of boys as part of the national immunization programme like US, Australia and Canada and some regions in Germany and Austria. In Australia for instance, HPV vaccination of all girls and boys is implemented through a routine school based programme.

In the base case result (i.e. vaccination of girls only, 85% vaccination rate) an ICER of 3,581 €/QALY is estimated when all HPV-related diseases are taken into account. Compared to some studies these are relative low ICERs. However, the results vary with the assumptions and the results are sensitive to the choice of discount rate, the choice of time horizon, vaccine price and vaccination rate. Seen in that perspective, the range of results should be considered. However, these Danish results estimating relative low ICERs is to some extent due to the high Danish incidence and mortality of HPV related cancers (e.g. the incidence and mortality from cervical cancer are among the highest in Europe, cf. Figure 3.3 and 3.4, page 33 in National Board of Health [27]). The incidence of genital warts is also relatively high in Denmark compared with other European countries [35] - resulting in a relatively large savings potential when a quadrivalent HPV vaccination program is introduced (the total treatment cost for genital warts is estimated to 8 mill. € per year [15]).

Comparison with results from others studies analysing HPV-vaccination in other countries is not straightforward because of differences in modelling approach (e.g., dynamic/agent-based versus cohort models) and other parameters, including vaccination scenario (2 or 3 doses) and other country specific conditions. However, the results for female vaccination only lie within the same range/level as the study from Elbasha et al. [18] – with the reservation that Elbasha et al. in principle include a catch-up programme (in the 2010-publication [15] it is shown that at catch-up programme increase the ICERs markedly). When including all HPV-related diseases in an US-setting Elabasha et al. estimate a mean ICER of 3232 $ per QALY gained in a scenario where girls and women 9-26 years of age were offered the vaccine [18]. Similarly, Chesson et al. in an US-setting estimated and ICER of 10500 $ per QALY gained given a 75% vaccination rate and vaccination of females aged 12-26 years [22].

On the other hand, Kim et al. [19], also in an US-setting, estimate an ICER of 18,130-20,990 $ per QALY gained in a scenario where 12-year old girls are vaccinated . The results are very sensitive to price.

Naturally, this analysis has a number of limitations. Firstly, only heterosexual transmission of HPV was simulated ignoring transmission between homosexual and heterosexual persons. Men who have sex with men (MSM) have a higher HPV disease burden (genital warts and anal cancers) and the current HPV programme will not protect this high-risk ground.

Secondly, unlike most other studies this model did not include naturally-acquired immunity to HPV in the simulations.

Thirdly, the model simulates a closed society with no continuous influx of unvaccinated persons. Increased globalisation increases the risk of HPV transmission across national borders thereby lowering the impact of potential the herd protection of men.

Fourth, 100% vaccine efficacy and lifelong protection was assumed. Assuming a reduced vaccine efficacy (99.8% or 93.5%) only change the results on the margin as shown in the 2010-publication [15]. On the other hand, inclusion of one booster dose (i.e. assuming that lifelong protection is not achievable) leads to an increase in the estimated ICERs (results not shown).

Fifth, it was assumed that the current Danish cervical screening program remained unchanged. Presently, the cervical screening program is being reviewed and it may in the long run be modified due to a decrease in the incidence of cervical cancer. Inclusion of a future modification of the screening program, leading to a possible reduction in the screening cost, in principle should be taken into account. However, it has not been taken into account leading to a marginal inaccurate estimation of future costs related to HPV-vaccination. Although sensitivity and scenario analyses are performed, health economic analyses using modelling is a simplified representation of real life involving several assumptions.

Sixth, two different sources were used for the applied QALY weights for the general population and for the specific occurrences/illnesses (Table 2). Furthermore, the QALY weights for the general population were distributed according to age group. This can lead to discrepancies. It appears from Table 2, for example, that the utility score for CIN1 was higher than any of the utility scores for the general female population, suggesting that the occurrence of CIN1 will not lead to any QALY loss. This is a limitation of the analysis, primarily caused by the use of two different sources for QALY weights. It is thus possible that the total gain in QALYs is underestimated in the present results.

Finally, possible future variations in the incidence of HPV-related cancers are not incorporated in the model simulations. The overall incidence of HPV related cancers has increased over the last 30 years in Denmark and the incidence of head and neck cancers is expected to increase further in the future. An underestimation of the future incidence leads to an underestimation of the cost of head and neck cancer.

Extension of the current HPV programme in Denmark to include boys and girls is a cost effective preventive intervention that would lead to a faster prevention of cancers, cancer precursors and genital warts in Denmark which is the country in the world with the highest risk of getting a cancer diagnosis [4].
